# An Experimentally Validated Structure-Based Virtual Screening Approach to Identify Nucleotide-Binding Protein Inhibitors as a New Source of Kinase Inhibitors

**DOI:** 10.3390/ph19081138

**Published:** 2026-07-23

**Authors:** Nicolas Bosc, Fabrice Carles, Jade Fogha, Blandine Baratte, Stéphane Bach, Samia Aci-Sèche, Stéphane Bourg, Sylvain Routier, Sandrine Ruchaud, Frédéric Buron, Pascal Bonnet

**Affiliations:** 1 Institut de Chimie Organique et Analytique (ICOA), UMR CNRS-Université d’Orléans 7311, Université d’Orléans BP 6759, 45067 Orléans Cedex 2, France; nicolas.bosc@univ-orleans.fr (N.B.); fabrice.carles@univ-orleans.fr (F.C.); jade.fogha@univ-orleans.fr (J.F.); samia.aci-seche@univ-orleans.fr (S.A.-S.); stephane.bourg@cnrs.fr (S.B.); sylvain.routier@univ-orleans.fr (S.R.); frederic.buron@univ-orleans.fr (F.B.); 2 Sorbonne Université, CNRS FR2424, Plateforme de criblage KISSf (Kinase Inhibitor Specialized Screening facility) Station Biologique, Place Georges Teissier, CS90074, 29688 Roscoff Cedex, France; baratte@sb-roscoff.fr (B.B.); bach@sb-roscoff.fr (S.B.); 3 Sorbonne Université, CNRS, UMR 8227, Integrative Biology of Marine Models, Station Biologique de Roscoff, CS 90074, 29688 Roscoff Cedex, France; sandrine.ruchaud@sb-roscoff.fr

**Keywords:** protein kinase, nucleotide-binding protein, virtual screening, haspin

## Abstract

**Background/Objectives:** Protein kinases represent major therapeutic targets because dysregulation of their phosphorylation activity is associated with several diseases, including cancer, diabetes, and inflammatory disorders. Thus, many researchers in the pharmaceutical filed are making significant effort to design potent new protein kinase inhibitors (PKIs) as potential drugs. In this context, we aimed to identify new alternatives by exploiting the chemical space defined by ligands of the nucleotide-binding protein family for the discovery of novel protein kinase inhibitors. Protein kinases bind the nucleotide adenosine triphosphate (ATP) and belong to the nucleotide-binding protein group. **Methods:** All ligands of the nucleotide-binding protein family, excluding known kinase inhibitors, that were identified in the ChEMBL database were used in a structure-based virtual screening approach. From this set, we aimed to identify novel nucleotide-binding protein inhibitors (NBPIs) as novel kinase inhibitors. A total of 19,709 NBPI compounds that were dissimilar to known PKIs were docked on five protein kinases, and the 200 best scoring docking poses were retained for potential purchase. **Results:** Only 25 compounds were commercially available in stock and were evaluated experimentally on a panel of 10 diverse protein kinases. Three NBPI compounds, one of which had originally been identified as active against the ATP-binding cassette transporter ABCG2, were identified as Haspin kinase inhibitors with micromolar activity. **Conclusions:** This study presents an efficient computational approach to identifying novel kinase inhibitors from a database of ligands of the nucleotide-binding protein family, and the protocol could be applied to other protein target families.

## 1. Introduction

Protein kinases are important components involved in biological processes like cell division, neuronal survival or insulin response [1,2,3]. Within the human proteome, about 2% belong to this superfamily, in which 518 protein kinases have been identified [4]. They are organized into groups and families such as the tyrosine kinase (TK) and Ca^2+^/calmodulin-dependent kinase (CAMK) groups, which are also divided into subfamilies. These proteins share a common phosphorylation mechanism involving the transfer of the γ-phosphate group of adenosine triphosphate (ATP) to the hydroxyl group on the substrates. ATP interacts with the hinge region between the N-lobe and the C-lobe of protein kinases [5].

In the case of aberrant phosphorylation, protein kinases are also related to various human disorders, including cancer [6], neuronal degeneration [7] and diabetes [8]. In fact, the protein kinase family was identified as a major drug target in the current century [9], and considerable efforts have been made to find or design protein kinase inhibitors (PKIs) as new potential drugs. To date, since the approval of imatinib in 2001, 100 small molecules have been approved by the US Food and Drug Administration (FDA) [10,11,12]. However, the development of protein kinase inhibitors faces important challenges, and only a small proportion of the kinome has been targeted so far. In addition, since drug resistance represents a major issue in kinase drug discovery, the discovery of new PKIs remains an active area of research.

ATP, the primary energy currency of the cell, is an abundant endogenous ligand of protein kinases and is not exclusively targeted by this superfamily. Indeed, numerous proteins involved in pivotal cellular processes include the ABC transporters, G-protein-coupled receptor superfamilies and phosphodiesterases. These proteins bind ATP or other nucleotides such as guanosine triphosphate (GTP) or cyclic adenosine monophosphate (cAMP) and are called nucleotide-binding proteins (NBPs). For many years, research activities oriented towards these proteins, including protein kinases, have established a collection of small molecules or inhibitors that can interact with the nucleotide site with interesting bioactivities. Based on similarities in protein structure, these nucleotide-binding protein inhibitors (NBPIs) could provide a new starting point for the discovery of novel series of ATP site-directed molecules. This approach appears particularly interesting because, as it has been demonstrated for the last few years, marketed drugs can also be repurposed for targeting protein kinases [13,14,15,16].

Most kinase inhibitors bind to the ATP binding site in the active conformation (type I and type I½) of protein kinases and in the inactive conformation (type II) but other inhibitors bind to an allosteric site close to the ATP-binding site (type III) or further away, such as the myristate site in BCR-Abl (type IV) [17].

Several studies have explored the identification of new kinase inhibitors, such as the quantitative structure–mutation–activity relationship tests (QSMART) [18], or by analyzing the growth inhibition profiles of 48 PKIs screened on 60 tumoral cell lines from the National Cancer Institute (NCI) [19]. More recently, a study combining network pharmacology and molecular docking validated experimentally the mechanism of naringenin in the context of cervical cancer treatment [20]. In another study, Dai et al. discovered novel nucleoside derivatives as lysine acetyltransferase p300 inhibitors with proliferation-inhibitory activity on cancer cells [21]. In this study, we sought to identify potential PKIs by exploring of the NBPI chemical library. We identified targets that share functional features with the kinome, using specific ontologies. Then, we collected from the ChEMBL database [22] inhibitors of these proteins which constituted the NBPI set, i.e., compounds that present an activity against NBP. A curated subset of these compounds was virtually screened on a selection of protein kinases using molecular docking and pharmacophore search. Finally, predicted hit compounds were experimentally validated on a panel of protein kinases to identify new PKIs.

## 2. Results and Discussion

**Figure 1:**
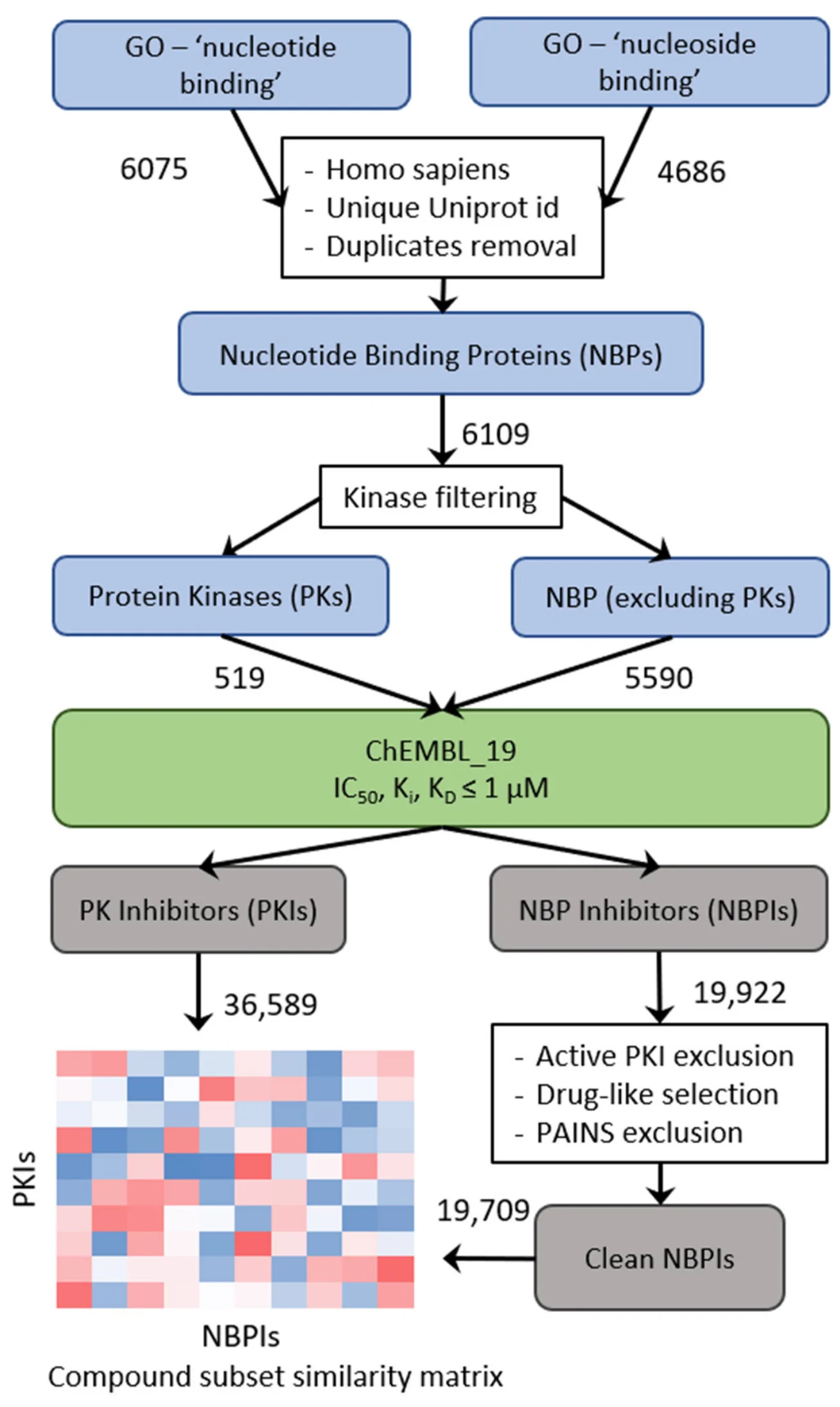
Schematic workflow representing the selection of compounds of the PKI and NBPI sets. Similarities between NBPIs and PKIs are color-coded between red (similar) and blue (dissimilar).

In organisms, the association of a nitrogenous base with a sugar forms a nucleoside, whereas the addition of phosphate groups produces a nucleotide. These are organic molecules that interact with nucleotide-binding proteins that serve as fuels for many biological functions. These proteins include the well-known protein kinases and others, such as ABC transporters and phosphodiesterases. In this study, protein kinases and other nucleotide-binding proteins were separated and analyzed independently. Thus, a nucleotide-binding protein set (excluding protein kinases—Table 1) was created, as well as a protein kinase dataset. Considering the link between nucleoside and nucleotide molecules, both sites were considered and will be referred as nucleotide-binding sites for simplification. As a result, 6075 and 4686 unique Uniprot identifiers were found with the nucleotide- or nucleoside-binding ontology tag, respectively. After the removal of duplicates, we split the remaining set of 6109 entries into a set of protein kinases (519) and a set of NBPs (5590). Using all these proteins, we identified and collected a set of 19,709 unique NBPIs and a set of 36,589 unique PKIs that have interesting bioactivity values (K_D_, IC_50_ or Ki below 1µM) available in the ChEMBL database (Figure 1).

Naturally, the NBPI set does not contain any protein kinase inhibitors with pIC50 > 6. Some filters were also applied to remove compounds that do not fulfill Lipinski’s rule of five or compounds with PAINS motifs.

**Figure 2:**
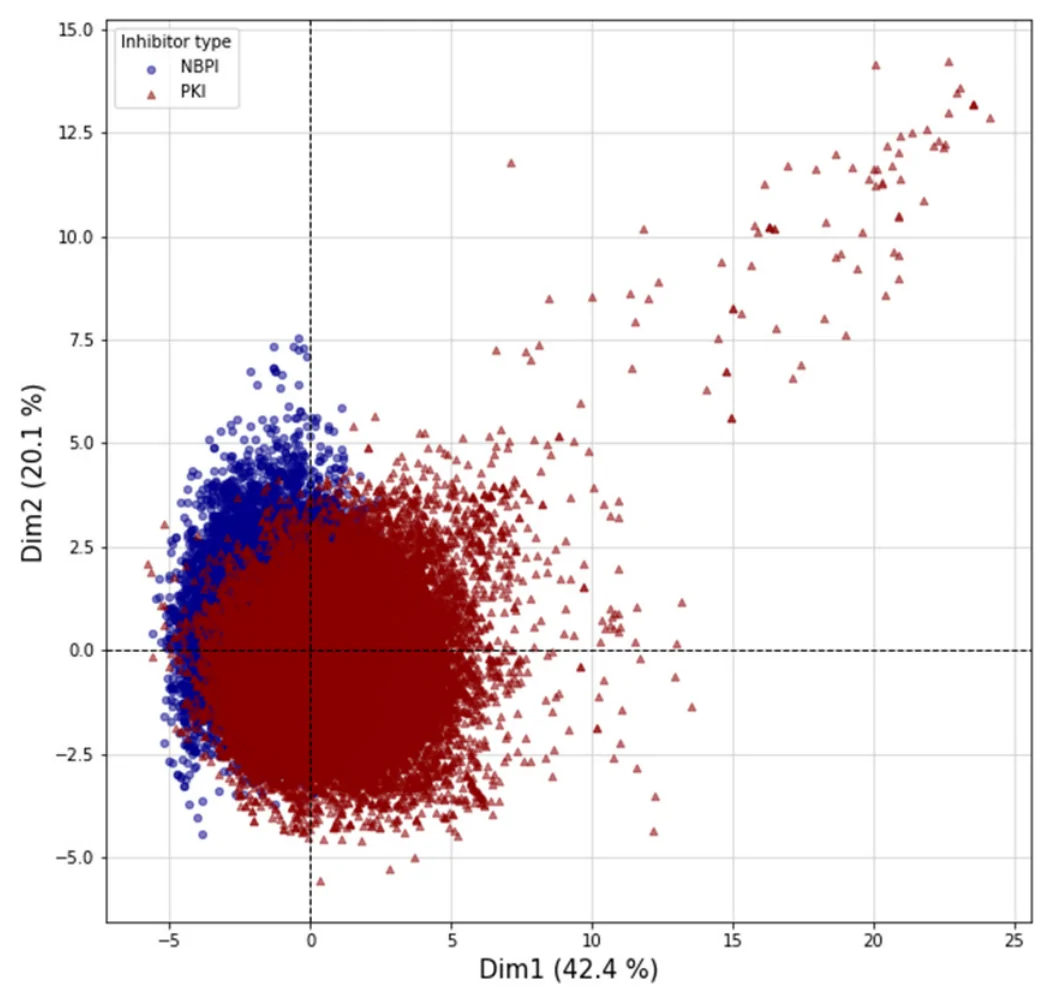
Principal component analysis (PCA) of NBPI (blue) and PKI (red) sets based on computed descriptors molecular weight, Wildman–Crippen topological partition coefficient LogP, number of hydrogen bond acceptors, number of hydrogen bond donors, topological polar surface area, number of rotatable bonds, number of aromatic rings, Labute’s approximate surface area, fraction of C atoms that are sp3 hybridized, molecular quantum numbers 8 and 10. PCA representation in two dimensions: Dim1 and Dim2 respectively explain 42.4% and 20.1% of the total variance.

**Figure 3:**
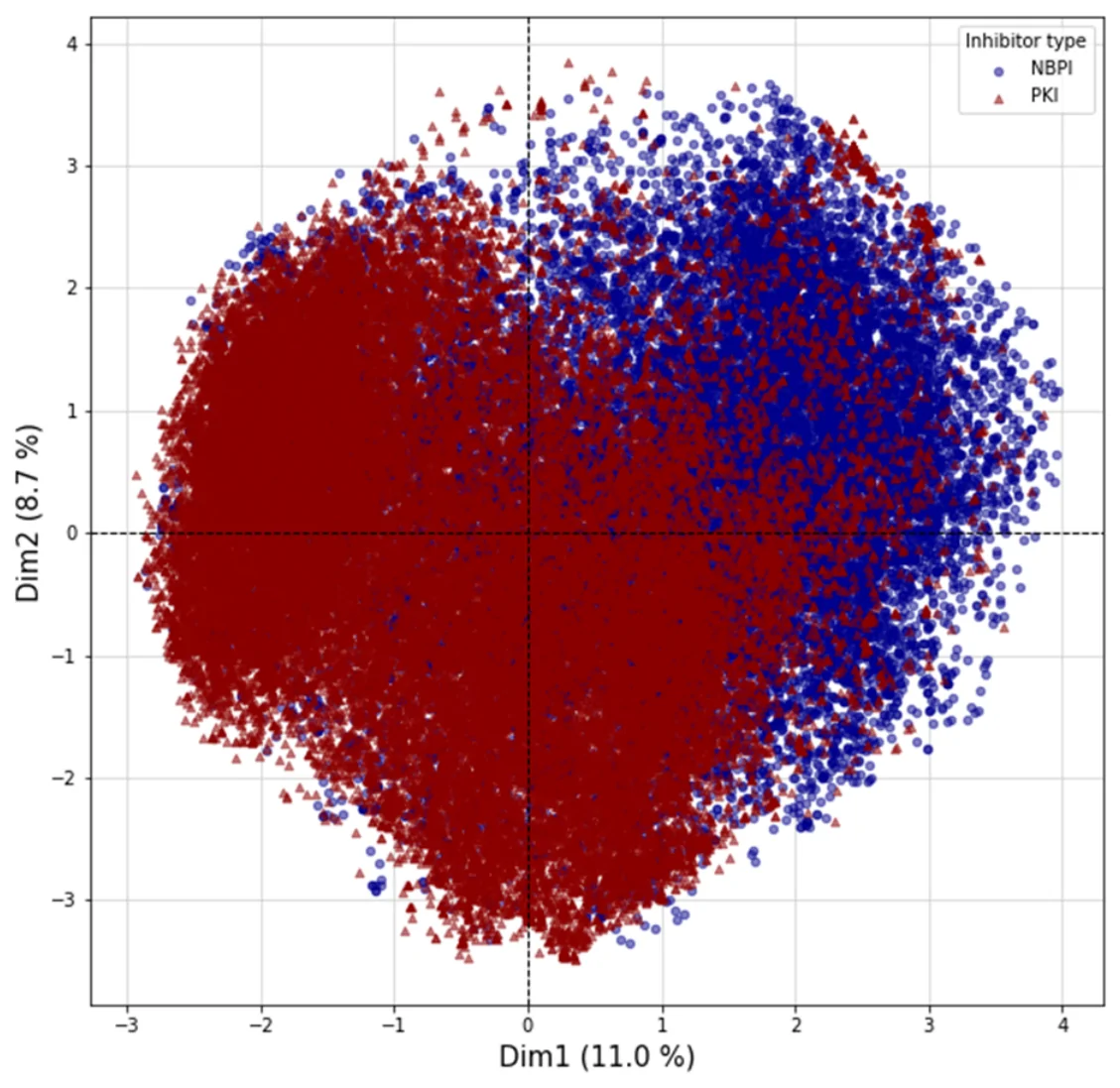
Principal component analysis (PCA) of NBPI (blue) and PKI (red) sets, based on MACCS fingerprints. PCA representation in two dimensions: Dim1 and Dim2 respectively explain 11.0% and 8.7% of the total variance.

In order to compare the NBPI and PKI sets, the different compounds were described using 11 physicochemical properties. Then, based on those descriptors, we represented the two sets in a reduced-dimensionality space through a principal component analysis (PCA). We found that the first two principal components explain 62.5% of the total variance (Figure 2). In order to analyze the impact of the molecular descriptors in the individuals factor map, we also used the MACCS fingerprints (Figure 3). In the representation, we observed that the main parts of NBPI and PKI compounds, colored in blue and red, respectively, could not be fully discriminated based on the selected descriptors (Figure 2 and Figure 3). Only a small part of the NBPI set was not mingled with the PKI one. This result suggests that compounds from the NBPI and PKI sets share a relatively similar chemical space. Hence, the NBPI dataset could be a promising source of novel PKIs.

### 2.1. Virtual Screening

Direct competition with ATP is an efficient approach to inhibit protein kinase function. Indeed, most described PKIs are able to bind the ATP site and to form strong interactions with the hinge region of protein kinases. Although for years, targeting the ATP site was considered as less selective than PKI targeting different protein kinase back pockets, several studies have shown that there are more important factors that could explain the promiscuity of PKIs [23].

To identify novel PKIs, we performed a structure-based virtual screening using five different protein kinases (AURKB, CDK2, CDK5, CLK1 and DYRK1A) in their active DFG-in conformation. In this context, we limited the search towards Type I kinase inhibitors. The protein structures were extracted from crystal structures of protein–ligand complexes retrieved from the PDB: AURKB (PDB entry 4AF3) [24], CDK2 (PDB entry 4EON) [25], CDK5 (PDB entry 3O0G) [26], CLK1 (PDB entry 2VAG) [27] and DYRK1A (PDB entry 2VX3) [28]. During the virtual screening process, ligands of the NBPI set were selected depending on their ability to correctly fit the ATP-binding site through molecular docking. As a result, different docking poses were positioned in the ATP-binding site of the different proteins. They were ranked by their Glide docking score. In a second step, a pharmacophore composed of a hydrogen bond acceptor located near the hinge region was created. Ligand poses matching this pharmacophore were conserved, and in the last step, the best poses of the 40 top-ranked docked compounds were kept for each target. Interestingly, several of the same compounds were found to bind with different kinases with high docking scores.

Finally, the virtual screening process identified 45 different compounds from the NBPI set that can interact with the hinge region of different protein kinases. Of these, 20, 13 and 12 compounds have a Tanimoto similarity coefficient (Tc) of Tc < 0.6, 0.6 ≤ Tc < 0.7 and Tc ≥ 0.7 to PKIs, respectively. To experimentally confirm these interactions, we purchased 25 of the available compounds from the Ambinter catalogue, a global supplier of chemical compounds (Table S1). Unfortunately, the compounds with the highest Glide score were not always available in commercial databases.

### 2.2. Kinase Assays

The 25 selected compounds were tested on a selectivity panel of protein kinases that belong to the CMGC group (CDK2, CDK5, CDK9, CLK1, DYRK1A), the CAMK group (PIM), the CK1 group (CSNK1A1 or CK1), the TKL group (RIPK3), and the other group (AURKB, Haspin). First, we evaluated the biological activity of the different compounds at 10 µM on each target (Table S1). For the compounds having more than 50% inhibition, IC_50_ values were determined along the same panel of protein kinases, and their corresponding pIC_50_ values were calculated. This corresponded to compounds **5**, **8**, **9**, **10**, **11**, **12**, **13** and **17** reported in Table 2. We obtained a hit rate of 0.05% among the 25 screened compounds. Focusing on pIC_50_ results reported in Table 2, only two compounds (**5** and **17**) could not be confirmed (false positives on the first screen at 10 µM single concentration) and were inactive on all protein kinases with a pIC_50_ value lower than 5. The other hit compounds showed different activity profiles. In fact, six compounds had pIC_50_ values that covered an interval from 5.2 to 6.5. It is important to mention that some of the compounds have low similarity to known kinase inhibitors available in the ChEMBL database, and no chemical optimization was performed on the compounds.

Moreover, a selectivity trend emerged from these biological results [29]. In Table 2, two compounds seem to be selective on the small kinase panel. Compound **9** has a low pIC_50_ value of 5.4 toward DYRK1A but is inactive (pIC_50_ < 5) for the other tested kinases. Quinoline is a heterocyclic scaffold that has been used in various kinase inhibitor drugs such as in bosutinib, anlotinib, lenvatinib, neratinib, tivozanib or cabozantinib [11]. Compound **11** is active on CDK9 with a low pIC_50_ value of 5.7, and inactive on the other cyclin-dependent kinases in our panel, CDK5 and CDK2, despite sharing similar kinase sequences [30]. However, it is important to mention that the activity for these two compounds is close to the threshold value (pIC_50_ of 5), so care has to be taken regarding the differences between active and inactive compounds. Compounds **8** and **10** also have interesting activity values for targets DYRK1A and CLK1 at almost a micromolar level (Table 2), and they are more specific for Haspin than for the other kinases. Lastly, compound **13**, with a pIC_50_ activity value of 6.4 on Haspin, is selective to this protein kinase given our kinase panel. To summarize, by screening all the compounds on the panel of protein kinases, we identified six relatively selective hit compounds (**8**, **9**, **10**, **11**, **12** and **13**) selected from the NBPI dataset. These compounds are active on the five protein kinases Haspin, DYRK1A, CLK1, CDK9 and AURKB.

**Figure 4:**
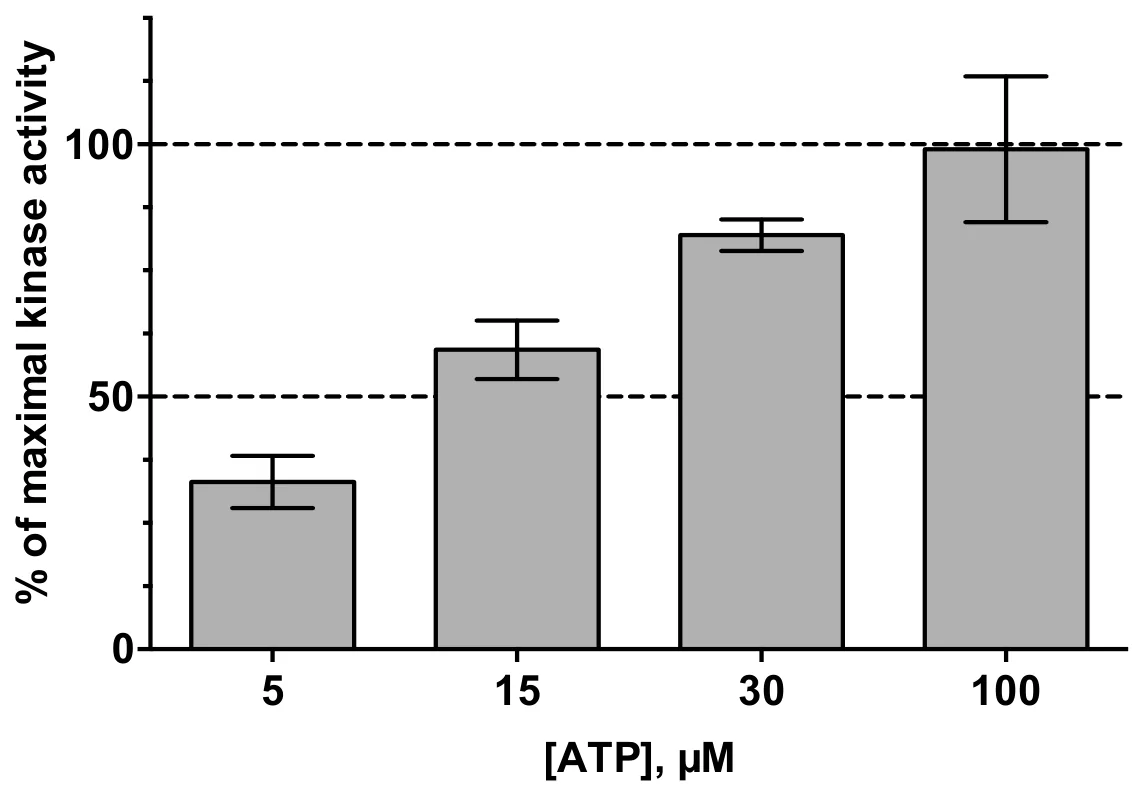
Haspin inhibition by compound **8**. Haspin kinase activities in the presence of 10 µM of compound **8** and increasing doses of ATP (from 5 to 100 µM). Data are mean (*n* = 3) ± SD.

Since compound **8** was the most active of all the compounds, we conducted further inhibition studies at 5, 15, 30 and 100 µM concentrations of ATP in triplicate (Figure 4). The inhibition of Haspin kinase activity by compound **8** was clearly modified by increasing concentrations of ATP. These data indicate that compound **8** is an ATP-competitive Haspin inhibitor.

**Figure 5:**
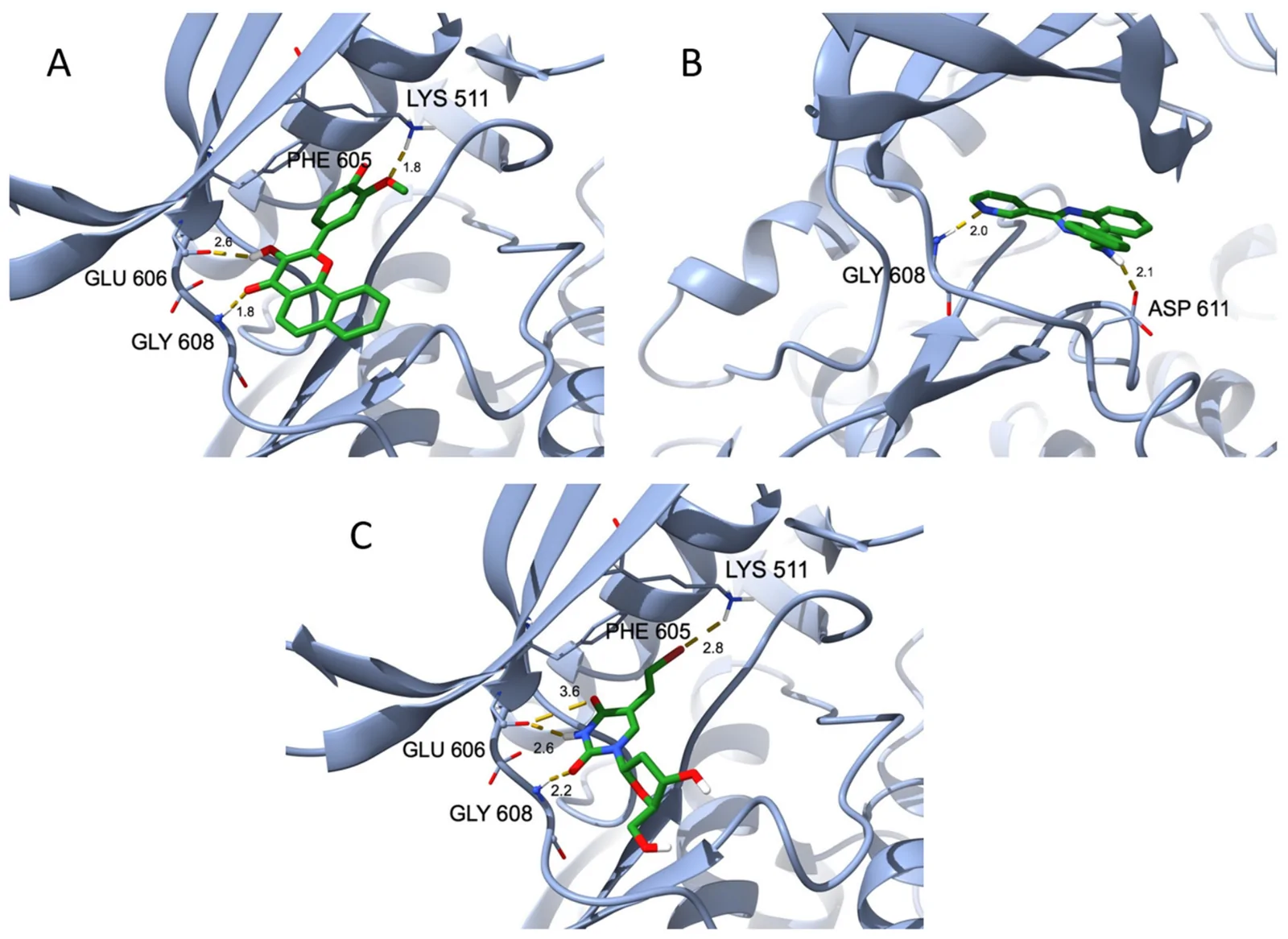
Predicted binding mode in the ATP site of Haspin (PDB entry 4OUC) for compounds having the best experimental activities, (**A**) **8**, (**B**) **10** and (**C**) **13**. Hydrogen bonds are represented in yellow dashed lines.

Interestingly, compounds **8**, **10** and **13** exhibit the highest activity values for Haspin kinase with a pIC_50_ of 6.5, 6.3 and 6.4, respectively, and good selectivity based on the small kinase panel, which could provide novel starting points for drug design projects. Compound **8** was originally designed against Breast Cancer Resistance Protein (BCRP/ABCG2), a member of the superfamily of ATP-binding cassette proteins [31]. To further investigate their specific interactions with Haspin kinase, docking experiments of these three compounds into the ATP site of Haspin were performed since this kinase was not selected in the initial virtual screening. All three compounds formed hydrogen bond interactions, see Figure 5, such as with Gly608 and Phe605 for compound **8**, Gly608 and Asp611 for compound **10** and Glu606, Gly608 and Asp611 for compound **13**. These residues are located in the hinge region of Haspin kinase. For compound **8**, we noticed an additional hydrogen bond interaction with the catalytic Lys511 that stabilizes the complex. Thus, these compounds have interesting potency and are predicted to interact and compete with ATP.

Finally, we estimated the chemical similarity of these compounds to known PKIs. Tanimoto similarity coefficients between NBPI and PKI compounds were assessed using MACCS molecular fingerprints, and the structure of the nearest PKI neighbor was retrieved. As shown in Table 3, the best confirmed hit compounds generally exhibited high similarity values (higher than 0.7). On the contrary, inactive compounds **5** and **17** had a similarity value of less than 0.6. Therefore, the hit compounds share structural similarity with known PKIs present in the ChEMBL database. This similarity may be limited to the same chemical groups or moieties or can be extended to a scaffold similar to that for compounds **10** and **11**. However, while there is a high similarity between compounds of the PKI and NBPI sets, these compounds might bind differently in protein kinases. For example, NBPI compound **10** is expected to bind to the hinge region through a hydrogen bond between Gly608 and the pyridine ring (Figure 4), and a strong analogue of its nearest PKI neighbor (CHEMBL102726) binds to the hinge region of CDK8 through the N-(4-quinazolinyl)amine moiety (PDB entry 6T41) [32]. While these compounds have a high Tc similarity value (Tc = 0.9), they bind differently to the kinase ATP site. Focusing on the nearest PKI neighbors, they do not show any activities for protein kinases present in our panel (Table S2). For instance, compound **10** presents additional alkyl and pyridine groups compared to its PKI neighbor (ChEMBL ID: CHEMBL102726—Table 3) but the latter is active on two kinases (EGFR and MAP kinase ERK2), as found in ChEMBL. These preliminary results demonstrate the interest in exploring novel chemical space close to the PKIs but also show that compounds dissimilar to PKIs have lower probability of being active on protein kinases.

It is important to note that the Tanimoto similarity coefficient between two compounds strongly depends on the calculated fingerprints. As shown in Table 3, the two compounds CHEMBL31634 and CHEMBL3116439 have a Tc of 0.70 and 0.36 with MACCS and RDKit topological fingerprints, respectively. MACCS fingerprints have the tendency to provide higher Tanimoto similarity coefficients. Even if the compounds with the highest similarity to known PKIs seem to present better potency, by using the RDKit topological fingerprint, we would have increased the chance of selecting compounds dissimilar to known kinase inhibitors.

While some molecules present reasonable biological activities on some protein kinases and good selectivity profiles on the small studied kinase panel, additional experiments are needed to advance these kinase inhibitor hits toward lead compounds, such as improving primary kinase activity, evaluating cellular activity and ADME-tox properties and performing selectivity assessment on a larger kinase panel.

## 3. Materials and Methods

### 3.1. Target Identification

To identify the nucleotide-binding proteins (NBPs), each human protein annotated as having a nucleoside- or nucleotide-binding site was selected using the gene ontology [33,34]. Using the web application AmiGO version 2.4 (http://amigo.geneontology.org/amigo/landing, accessed on 1 September 2016), we searched for all entries associated with the terms ‘nucleotide binding’, respectively ‘nucleoside binding’, and downloaded only those associated with a human Uniprot identifier [35,36].

### 3.2. Compound Collections

Using the set of NBPs described in the previous section, we searched for compounds that had been experimentally tested against this panel. The ChEMBL database (version 19) [22] interfaced through the pipelining software KNIME 2.9 [37] was used to extract all the bioactivities associated with each of these proteins and retained compounds with a K_D_ (dissociation constant), Ki (inhibition constant) or IC_50_ (Half-maximal inhibitory concentration) below 1µM, resulting in a set of 19,922 unique compounds. For this study, we removed all nucleotide-binding protein inhibitors (NBPIs) that had previously been tested against any protein kinase to focus exclusively on previously unexplored compounds (from a ChEMBL perspective). Then, the negative logarithms of these values (pK_D_, pK_i_ and pIC_50_) were used in the study [38].

Additionally, we applied some drug-like filters [39,40] to preferentially select compounds of the NBPI set with an acceptable ADME profile that corresponds to compounds having a number of non-hydrogen atoms greater than 5, a molecular weight between 200 and 500 Da, a topological polar surface area that does not exceed 140 Å^2^, no more than 10 rotatable bonds, less than 10 hydrogen bond acceptors, less than 5 hydrogen bond donors and a lipophilicity (logP(o/w)) of less than 5. Moreover, we removed compounds that contained non-organic atoms or reactive functional groups. All the molecular descriptors have been calculated using MOE (Molecular Operating Environment, Chemical Computing Group Inc., Montreal, QC, Canada) [41]. Finally, we removed Pan-Assay Interference Compounds (PAINS) using a KNIME workflow published previously [42].

### 3.3. Dataset Comparison

Two principal component analyses (PCAs) were performed on the compounds of the NBPI and PKI sets with sklearn [43], a python machine learning package. PKIs with MW > 1000 Da were removed before PCA calculations. It is important to note that almost half of FDA-approved PKIs fail to satisfy at least one descriptor of Lipinski’s rule of five [44]. For the purpose of the first PCA, 11 descriptors describing the chemical space of kinase inhibitors [10] were calculated for each compound: molecular weight, Wildman–Crippen topological partition coefficient LogP, number of hydrogen bond acceptors, number of hydrogen bond donors, topological polar surface area, number of rotatable bonds, number of aromatic rings, Labute’s approximate surface area, fraction of C atoms that are sp3 hybridized, molecular quantum numbers 8 and 10 (Figure 2). For the purpose of the second PCA, MACCS (Molecular ACCess System) structural key fingerprints were calculated (Figure 3). All descriptors were calculated using the RDKit package [45].

### 3.4. Compound Similarity

Using MACCS molecular fingerprints, we calculated the pairwise similarity between the NBPI and PKI datasets using the Tanimoto coefficient (Tc). For each compound of the NBPI set, we retrieved the nearest PKI neighbor according to the highest Tc value. Bioactivity values and kinase targets associated with these nearest PKI neighbors were retained.

### 3.5. Virtual Screening

A structure-based virtual screening campaign was performed in two different steps: molecular docking and pharmacophore search using Schrodinger Molecular Modeling Suite 2013 update 2 [46].

We retrieved the crystal structures from the protein data bank (PDB) [47] of the following protein kinases, which were being studied internally as part of a drug design project involving medicinal chemists: AURKB (PDB entry 4AF3; DFG-in) [24], CDK2 (PDB entry 4EON; DFG-in) [25], CDK5 (PDB entry 3O0G; DFG-in) [26], CLK1 (PDB entry 2VAG; DFG-in) [27] DYRK1A (PDB entry 2VX3; DFG-in) [28]. The conformational state of the DFG motif is specified for each complex. In each PDB file, the subunit A was selected, except for PDB entry 3O0G structure (CDK5), where the subunit B, crystalized with a ligand, was used. For the CDK5 kinase, the Asn144 in the DFG motif was mutated in Asp144 since it has been mutated in the crystal structure. Protein kinase structures were prepared using the Protein Preparation Wizard workflow of the Schrodinger Molecular Modeling Suite [48]. For each protein, we performed the following preprocessing steps (hydrogen atoms added, incomplete residues filled); bond orders and the ligand connections were manually corrected. Exhaustive sampling was performed for hydrogen-bond assignment, and complexes were finally refined by a minimization stage with a constraint RMSD of 0.3 Å to converge to a final structure using OPLS2005 force field, essentially to remove steric clashes. The docking grid used for the compounds of the NBPI set was calculated according to the position of the ligand located in the active site of the respective protein kinase. Docking calculations were performed using the Glide module with extra precision [49,50], ligand flexibility was taken into account, and the sampling of ring conformation option was activated. In total, 20 poses per ligand were generated.

A pharmacophore model was generated using the staurosporine ligand anchored to the aligned active sites of the different kinases (Figure S1). As a pharmacophore feature, we selected the hydrogen bond acceptor of the carbonyl group of the pyrrolone moiety close to the hinge region with a radius of 2 Å. Docking poses were filtered using this pharmacophore. Based on the docking scores, 25 compounds among the top 200 poses were purchased because of their availability in stock at AMBINTER (http://www.ambinter.com/) and then experimentally validated on a panel of kinases.

### 3.6. Post-Processing Docking

The hit compounds were docked a second time on other kinases available in the experimental panel using the Glide module of Schrodinger Molecular Modeling Suite 2013 [49,50] with the same parameters as described previously. Additional protein kinases extracted from PDB were CDK9 (PDB entry 3BLR) [51], CK1 or CSNK1A1 (PDB entry 5FQD) [52], Haspin (PDB entry 4OUC) [53], and PIM1 (PDB entry 1XWS) [unpublished data]. Twenty poses per ligand were generated.

### 3.7. Kinase Assay

Kinase activities were assayed using either a protein or a peptide as the substrate in the presence of 15 µM [γ-^33^P] ATP (3000 Ci/mmol; 10 mCi/mL), in a final volume of 30 µL following the assay described in [54]. Controls were performed using appropriate DMSO dilutions (dimethylsulfoxide). Full-length kinases were used unless otherwise specified. Peptide substrates were obtained from Proteogenix (Oberhausbergen, France). To determine half-maximal inhibitory concentrations (IC_50_), experiments were performed in duplicate using serial dilutions of the compounds under investigation. Dose–response relationships were generated for each compound, and IC_50_ values were calculated by nonlinear regression (variable-slope model) of dose–response curves obtained from 10 serial concentrations tested in duplicate using GraphPad Prism software (GraphPad Software version 8.0.0, San Diego, CA, USA). The reliability of the curve fitting was evaluated using the coefficient of determination, with only datasets exhibiting R^2^ values greater than 0.85 being considered acceptable. Enzymatic activities measured in the presence of inhibitors were normalized relative to the maximal activity recorded in the absence of inhibitor, and results were expressed as percentages of the control activity. The following kinase buffers (A–R) were used: (A) 10 mM MgCl_2_, 1 mM EGTA, 1 mM DTT (dithiothreitol), 25 mM Tris-HCl pH 7.5, 50 µg/mL heparin; (B) 60 mM β-glycerophosphate, 30 mM p-nitrophenyl-phosphate, 25 mM MOPS (3-(N-morpholino)propanesulfonic acid) (pH 7), 5 mM EGTA, 15 mM MgCl_2_, 1 mM DTT, 0.1 mM sodium orthovanadate; (D) 25 mM MOPS, pH 7.2, 12.5 mMβ-glycerophosphate, 25 mM MgCl_2_, 5 mM EGTA, 2 mM EDTA, 0.25 mM DTT; (H) MOPS 25 mM pH 7.5, 10 mM MgCl_2_; (R) 5 mM MOPS pH 7.2, 2.5 mM β-glycerophosphate, 4 mM MgCl_2_, 2.5 mM MnCl_2_, 1 mM EGTA, 0.4 mM EDTA, 50 μg/mL BSA, 0.05 mM DTT.

Aurora B (human, expressed in Sf9 cells, SignalChem, Richmond, BC, Canada, product #A31-10G) was assayed in buffer D with 0.2 µg/µL of Myelin-Basic Protein (MBP) as substrate. CDK2/CyclinA (human cyclin-dependent kinase-2, kindly provided by Dr. A. Echalier-Glazer, Leicester, UK) was assayed in buffer A (supplemented with 0.15 mg/mL of Bovine Serum Albumine, BSA, and 0.23 mg/mL of DTT) with 0.8 µg/µL of histone H1 as substrate. CDK5/p25 (human, expressed in bacteria) was assayed in buffer B, with 0.8 µg/µL of histone H1 as substrate. CDK9/CyclinT (human, recombinant, expressed by baculovirus in Sf9 insect cells) was assayed in buffer A (supplemented with 0.15 mg/mL of BSA and 0.23 mg/mL of DTT) with 0.27 µg/µL of the following peptide: YSPTSPSYSPTSPSYSPTSPSKKKK, as substrate. *Ssc*CK1δ/ε (casein kinase 1δ/ε, native, affinity purified from porcine brain extracts, *Sus scrofa domesticus*) was assayed in buffer B, with 0.022 µg/µL of the following peptide: RRKHAAIGSpAYSITA as CK1-specific substrate. *Mm*CLK1 (Cdc2-like kinase from *Mus musculus*, recombinant, expressed in bacteria) was assayed in buffer A (supplemented with 0.15 mg/mL of BSA and 0.23 mg/mL of DTT) with 0.027 μg/µL of the following peptide: GRSRSRSRSRSR. *Rn*DYRK1A (dual-specificity tyrosine phosphorylation-regulated kinase 1A from *Rattus norvegicus*, amino acids 1 to 499 including the kinase domain, recombinant, expressed in bacteria, DNA vector kindly provided by Dr. W. Becker, Aachen, Germany) was assayed in buffer A (supplemented with 0.5 mg/mL of BSA and 0.23 mg/mL of DTT) with 0.033 μg/µL of the following peptide: KKISGRLSPIMTEQ as substrate. Haspin (human, amino acids 470 to 798 including the kinase domain, expressed in bacteria) was assayed in buffer H with 0.007 µg/µL of Histone H3 peptide (aa 1–21) as substrate. PIM1 (human proviral integration site for Moloney murine leukemia virus kinase, expressed in bacteria) was assayed in buffer B with 0.8 µg/µL of histone H1 (Sigma #H5505, Sigma-Aldrich Corporation, St. Louis, MO, USA) as substrate. RIPK3 (human receptor interacting protein kinase 3, expressed in Sf9 cells) was assayed in buffer R with 0.1 µg/µL of MBP as substrate.

## 4. Conclusions

In the drug discovery process, protein kinases have been extensively studied, and their inhibitors occupy a highly crowded chemical space. As the necessity to discover new PKIs is still important, we propose a novel source of kinase inhibitors from existing nucleotide-binding protein inhibitors based on their common mode of binding in nucleotide or nucleoside active sites. Using a virtual screening protocol, a set of NBP compounds was predicted to bind the ATP active site of various protein kinases. While the most interesting NBPIs were not commercially available, we experimentally screened some predicted active compounds of a panel of kinases. We identified five compounds, in particular, that have moderate potencies on Haspin, DYRK1A, CLK1, CDK9 and/or AURKB kinases. In particular, the benzoflavone compound, originally designed against BCRP/ABCG2, is an ATP-competitive inhibitor of Haspin (pIC_50_ of 6.5) inducing a dose-dependent inhibition effect. The identified hits could be optimized further in drug discovery projects in order to increase their activities and ADME-tox properties. Hence, we expect our approach to open the way to unexplored chemical space. Finally, we show here that virtual screening of ligands bound to proteins that share similar putative substrates is an effective way to identify novel scaffolds.

## Figures and Tables

**Table 1:** Enumeration of the number of Uniprot IDs and the number of compounds associated with distinct nucleotide-binding protein classes, as reported in the ChEMBL database. A total of 26 UniProt identifiers were not associated with a protein class. A total of 19,847 compounds were tested on more than one target.

Protein Class	Number of Uniprot IDs	Number of Retrieved Compounds
Enzyme	136	11,635
Epigenetic regulator	5	126
Ion channel	16	3044
Membrane receptor	3	2988
Other cytosolic protein	4	1063
Structural protein	12	83
Transcription factor	1	160
Transporter	14	560
Unclassified protein	26	188

**Table 2:** Selectivity profile of the eight compounds confirmed on the kinase panel. pIC_50_ > 5 are colored in green. When available, molecule names are added below the chemical structure.

ID	Structure	AURKB	CDK2	CDK5	CDK9	CK1	CLK1	DYRK1A	HASPIN	PIM1	RIPK3
5	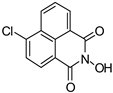	<5	<5	<5	<5	<5	<5	<5	<5	<5	<5
8	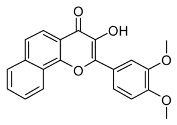	<5	<5	<5	5.5	<5	5.6	5.4	6.5	<5	<5
9	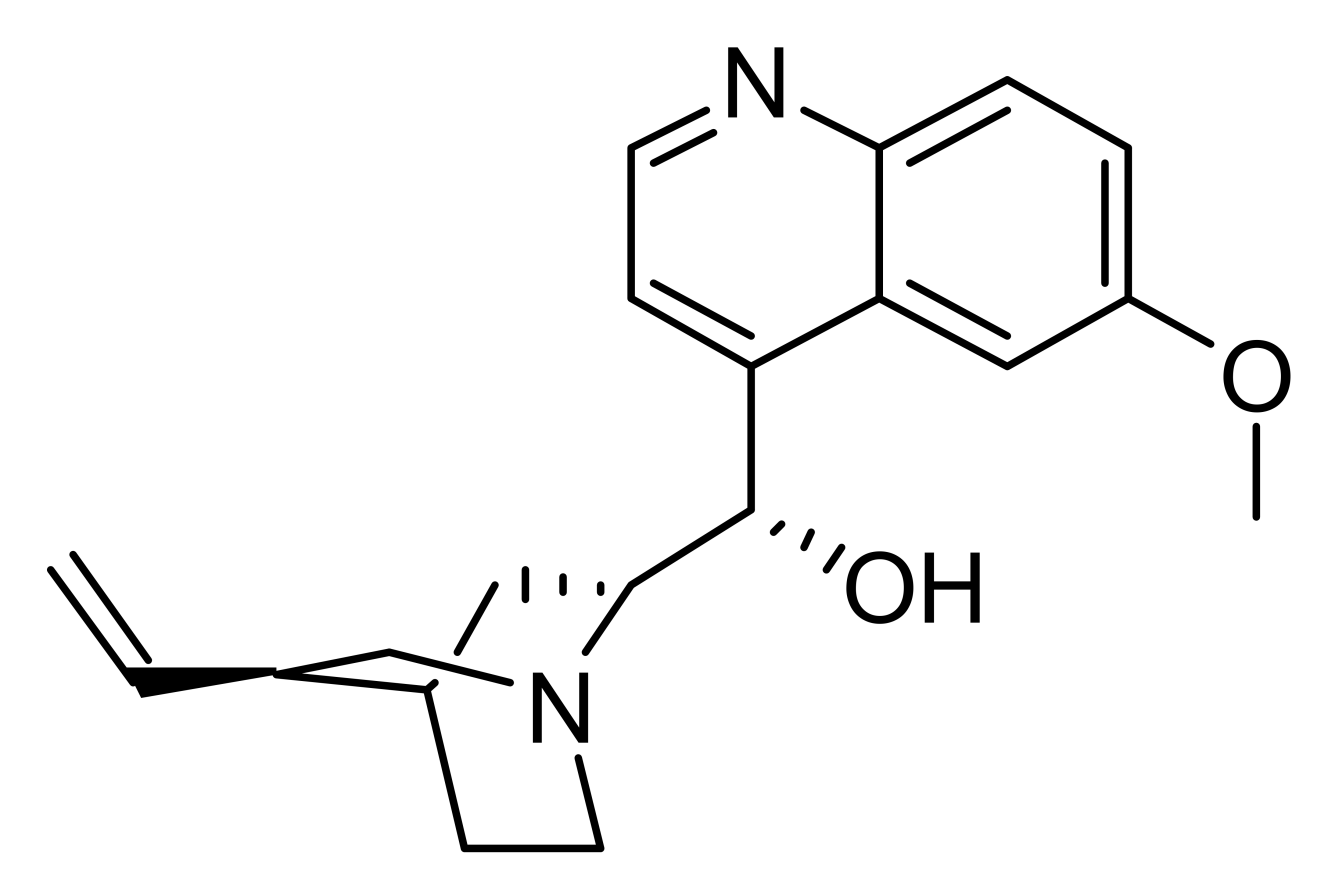 quinidine	<5	<5	<5	<5	<5	<5	5.4	<5	<5	<5
10	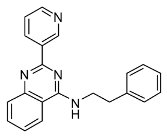	<5	<5	<5	<5	<5	5.6	5.7	6.3	5.2	<5
11	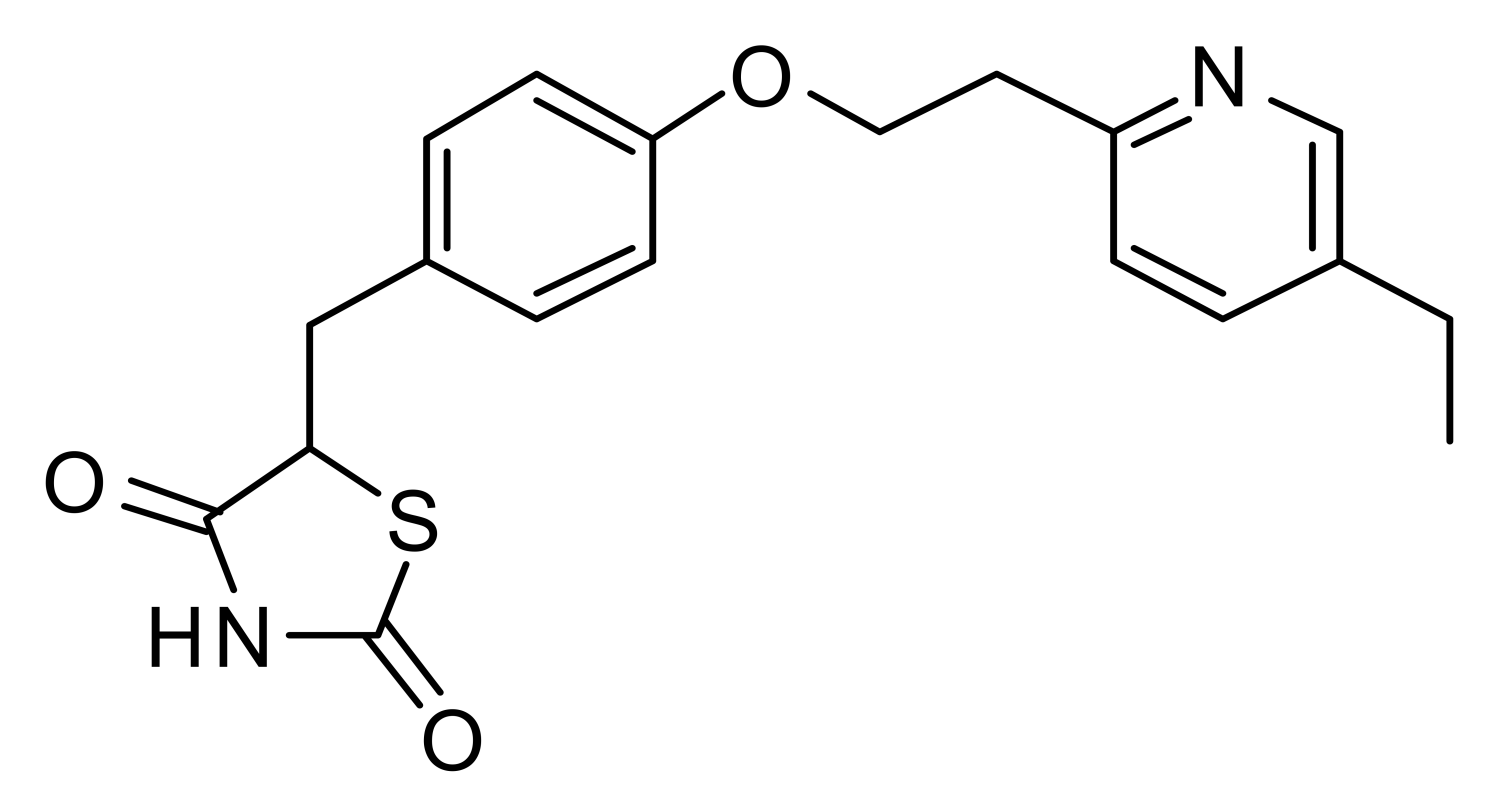 Pioglitazone	<5	<5	<5	5.7	<5	<5	<5	<5	<5	<5
12	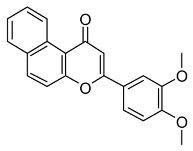	5.6	<5	<5	<5	<5	5.7	<5	<5	<5	<5
13	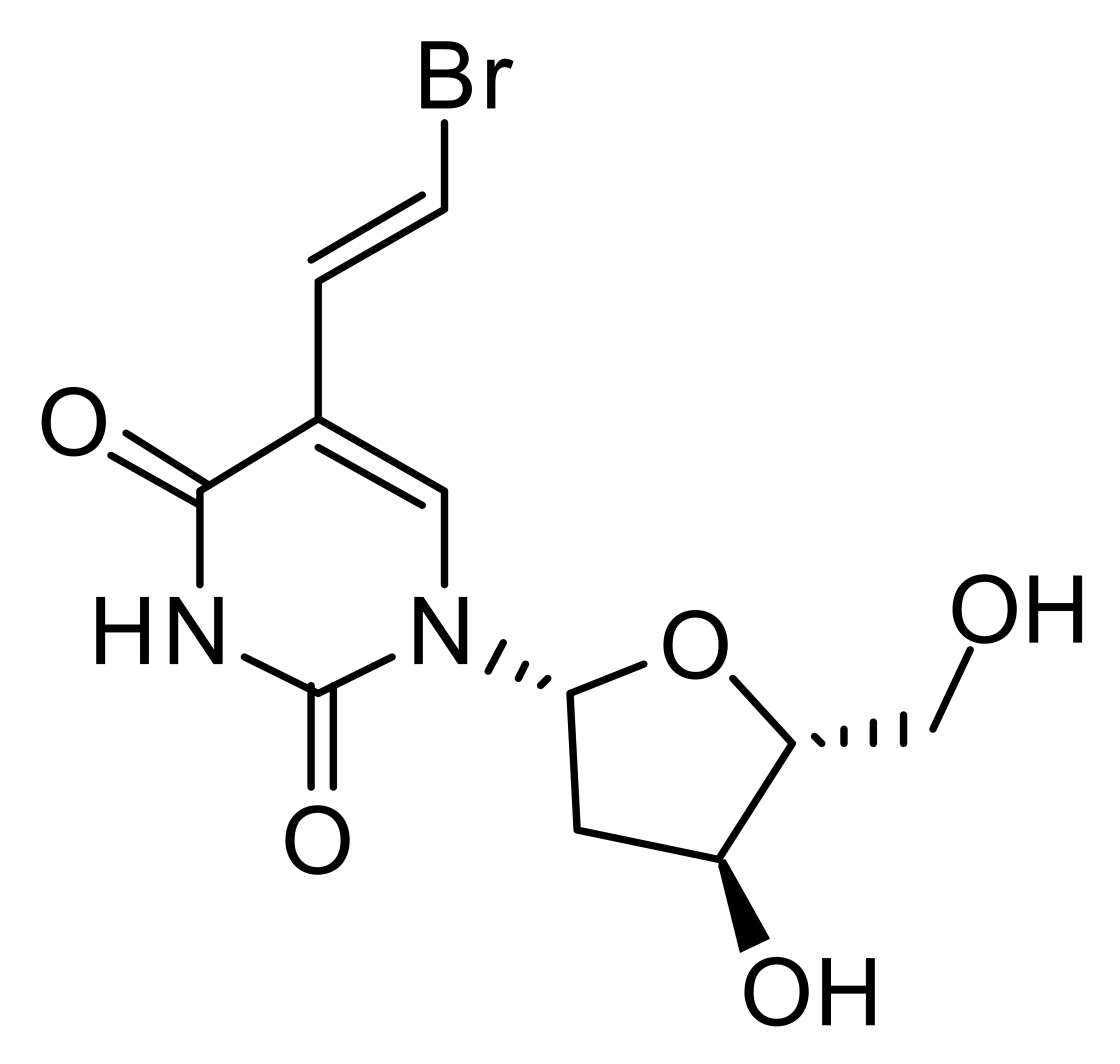 Brivudine	<5	<5	<5	<5	<5	<5	<5	6.4	<5	<5
17	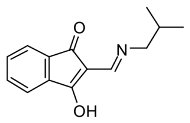	<5	<5	<5	<5	<5	<5	<5	<5	<5	<5

**Table 3:** Tanimoto similarity values using MACCS fingerprints between the eight screened compounds and their corresponding nearest PKI neighbors. In parenthesis, Tanimoto similarity values using RDKit topological fingerprints. Active compounds are shown in gray.

ID	Hit Compound	Nearest PKI Neighbor	Tanimoto Similarity Coefficient
8	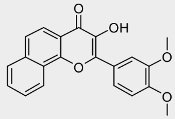 CHEMBL2420087	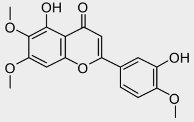 CHEMBL487402	0.94 (0.71)
9	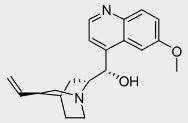 CHEMBL21578	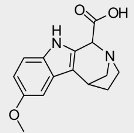 CHEMBL392876	0.77 (0.49)
10	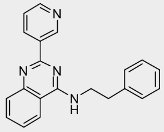 CHEMBL544818	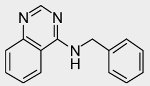 CHEMBL102726	0.86 (0.53)
11	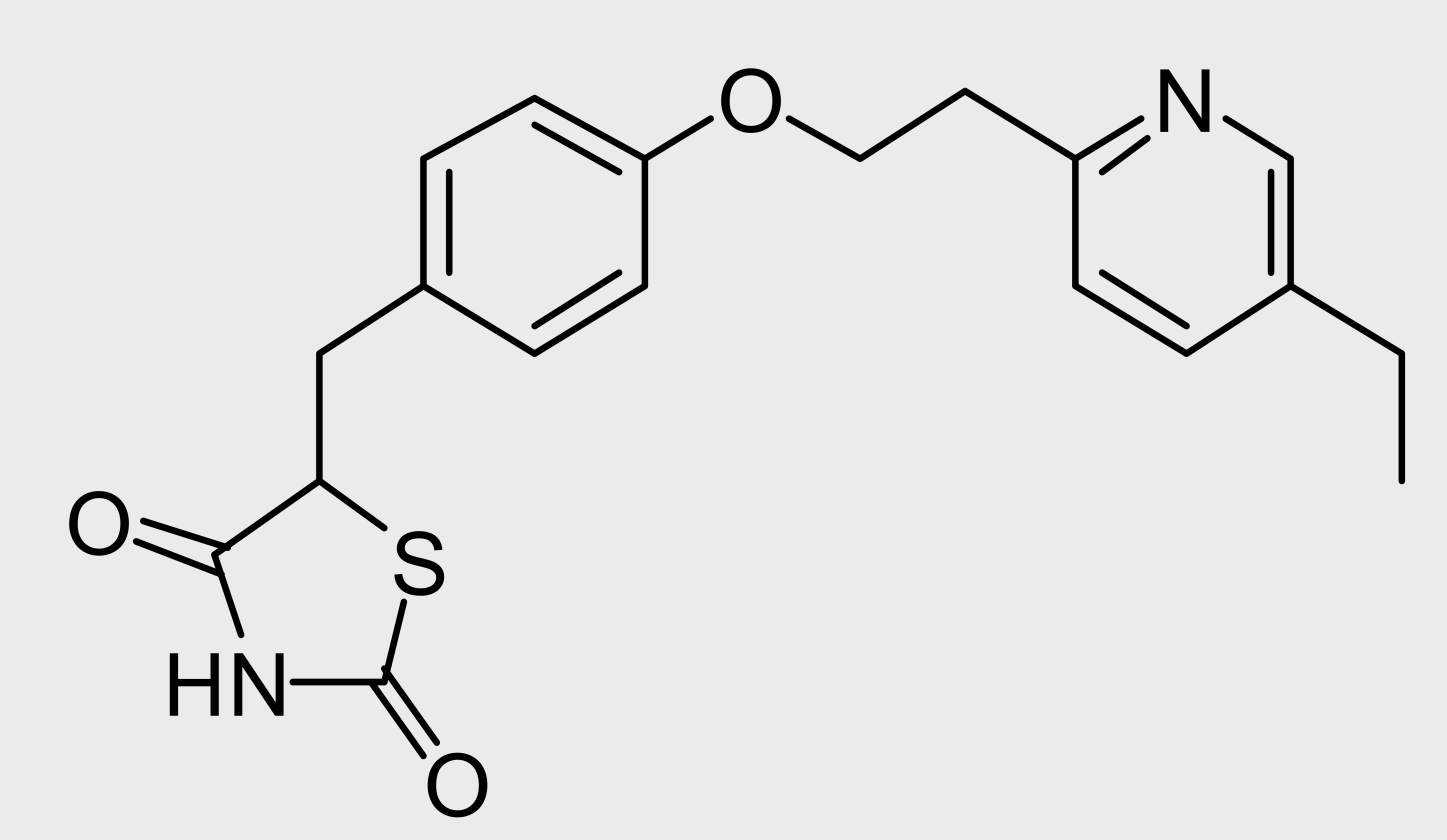 CHEMBL595	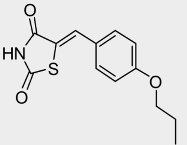 CHEMBL84558	0.77 (0.32)
12	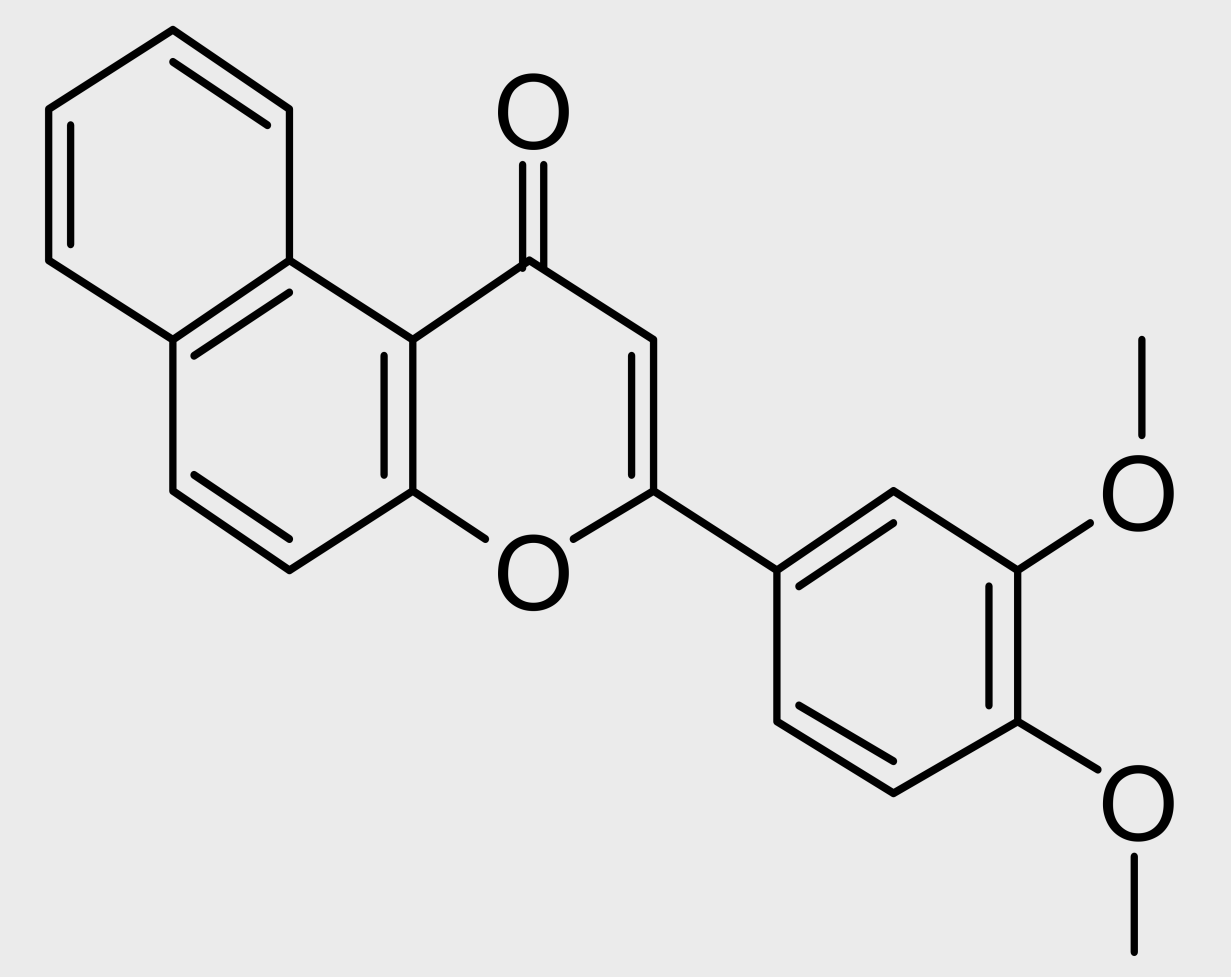 CHEMBL2420094	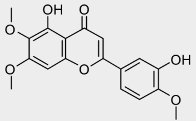 CHEMBL487402	0.91 (0.71)
13	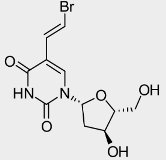 CHEMBL31634	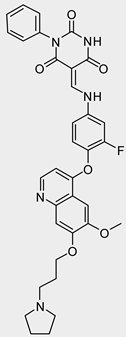 CHEMBL3116439	0.70 (0.36)
5	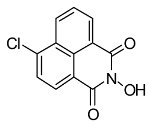 CHEMBL1415852	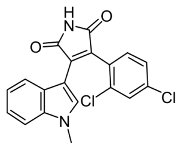 CHEMBL102714	0.56 (0.27)
17	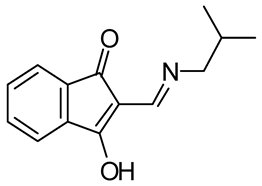 CHEMBL1982433	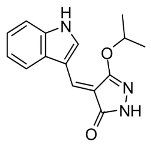 CHEMBL1989029	0.49 (0.30)

## Data Availability

The original contributions presented in this study are included in the article and supplementary material. Further inquiries can be directed to the corresponding author.

## Supplementary Materials

The following supporting information can be downloaded at: https://www.mdpi.com/article/10.3390/ph19081138/s1, Figure S1: Pharmacophore designed for virtual screening; Table S1: Percentage of remaining activity of 25 compounds obtained from the virtual screening for 10 protein kinases; Table S2: Biological activity data of the nearest PKI neighbors attached to the different hits, compounds **8**, **9**, **10**, **11**, **12** and **13**.

## Abbreviations

ATP	Adenosine triphosphate
CAMK	Ca^2+^/calmodulin-dependent kinase
cAMP	Cyclic adenosine monophosphate
CK1	Casein Kinases
CMGC	Cyclin-dependent kinases (CDKs), mitogen-activated protein kinases (MAP kinases), glycogen synthase kinases (GSK) and CDK-like kinases
FDA	US Food and Drug Administration
GTP	Guanosine triphosphate
NBP	Nucleotide-binding proteins
NBPI	Nucleotide-binding protein inhibitors
PKI	Protein kinase inhibitors
TK	Tyrosine kinases
TKL	Tyrosine kinase-like kinases

## References

[1] Nigg E.A. (2001). Mitotic kinases as regulators of cell division and its checkpoints. Nat. Rev. Mol. Cell Biol..

[2] Fukunaga K., Miyamoto E. (1998). Role of MAP kinase in neurons. Mol. Neurobiol..

[3] Nesher R., Anteby E., Yedovizky M., Warwar N., Kaiser N., Cerasi E. (2002). β-Cell Protein Kinases and the Dynamics of the Insulin Response to Glucose. Diabetes.

[4] Manning G., Whyte D.B., Martinez R., Hunter T., Sudarsanam S. (2002). The Protein Kinase Complement of the Human Genome. Science.

[5] Modi V., Dunbrack R.L. (2019). Defining a new nomenclature for the structures of active and inactive kinases. Proc. Natl. Acad. Sci. USA.

[6] Lapenna S., Giordano A. (2009). Cell cycle kinases as therapeutic targets for cancer. Nat. Rev. Drug Discov..

[7] Chiba T., Yamada M., Aiso S. (2009). Targeting the JAK2/STAT3 axis in Alzheimer’s disease. Expert Opin. Ther. Targets.

[8] Saltiel A.R., Kahn C.R. (2001). Insulin signalling and the regulation of glucose and lipid metabolism. Nature.

[9] Cohen P. (2002). Protein kinases—The major drug targets of the twenty-first century?. Nat. Rev. Drug Discov..

[10] Carles F., Bourg S., Meyer C., Bonnet P. (2018). PKIDB: A Curated, Annotated and Updated Database of Protein Kinase Inhibitors in Clinical Trials. Molecules.

[11] Bournez C., Carles F., Peyrat G., Aci-Sèche S., Bourg S., Meyer C., Bonnet P. (2020). Comparative Assessment of Protein Kinase Inhibitors in Public Databases and in PKIDB. Molecules.

[12] Mullard A. (2025). FDA approves 100th small-molecule kinase inhibitor. Nat. Rev. Drug Discov..

[13] Ashburn T.T., Thor K.B. (2004). Drug repositioning: Identifying and developing new uses for existing drugs. Nat. Rev. Drug Discov..

[14] Homan K.T., Larimore K.M., Elkins J.M., Szklarz M., Knapp S., Tesmer J.J.G. (2015). Identification and Structure--Function Analysis of Subfamily Selective G Protein-Coupled Receptor Kinase Inhibitors. ACS Chem. Biol..

[15] Park H., Hong S., Hong S. (2012). Nocodazole is a High-Affinity Ligand for the Cancer-Related Kinases ABL, c-KIT, BRAF, and MEK. ChemMedChem.

[16] Wan X., Zhang W., Li L., Xie Y., Li W., Huang N. (2013). A New Target for an Old Drug: Identifying Mitoxantrone as a Nanomolar Inhibitor of PIM1 Kinase via Kinome-Wide Selectivity Modeling. J. Med. Chem..

[17] Arter C., Trask L., Ward S., Yeoh S., Bayliss R. (2022). Structural features of the protein kinase domain and targeted binding by small-molecule inhibitors. J. Biol. Chem..

[18] Huang L.C., Yeung W., Wang Y., Cheng H., Venkat A., Li S., Ma P., Rasheed K., Kannan N. (2020). Quantitative Structure–Mutation–Activity Relationship Tests (QSMART) model for protein kinase inhibitor response prediction. BMC Bioinform..

[19] Ion G.N.D., Nitulescu G.M. (2020). In Search of Outliers. Mining for Protein Kinase Inhibitors Based on Their Anti-Proliferative NCI-60 Cell Lines Profile. Molecules.

[20] Zhou J., Li H., Wu B., Zhu L., Huang Q., Guo Z., He Q., Wang L., Peng X., Guo T. (2024). Network pharmacology combined with experimental verification to explore the potential mechanism of naringenin in the treatment of cervical cancer. Sci. Rep..

[21] Dai Q., Yuan Z., Sun Q., Ao Z., He B., Jiang Y. (2024). Discovery of novel nucleoside derivatives as selective lysine acetyltransferase p300 inhibitors for cancer therapy. Bioorg. Med. Chem. Lett..

[22] Zdrazil B., Felix E., Hunter F., Manners E.J., Blackshaw J., Corbett S., de Veij M., Ioannidis H., Mendez Lopez D., Mosquera J.F. (2023). The ChEMBL Database in 2023: A drug discovery platform spanning multiple bioactivity data types and time periods. Nucleic Acids Res..

[23] Hanke T., Mathea S., Woortman J., Salah E., Berger B.-T., Tumber A., Kashima R., Hata A., Kuster B., Müller S. (2022). Development and characterization of Type I, Type II, and Type III LIM-kinase chemical probes. J. Med. Chem..

[24] Elkins J.M., Santaguida S., Musacchio A., Knapp S. (2012). Crystal Structure of Human Aurora B in Complex with INCENP and VX-680. J. Med. Chem..

[25] Echalier A., Cot E., Camasses A., Hodimont E., Hoh F., Jay P., Sheinerman F., Krasinska L., Fisher D. (2012). An Integrated Chemical Biology Approach Provides Insight into Cdk2 Functional Redundancy and Inhibitor Sensitivity. Chem. Biol..

[26] Ahn J.S., Radhakrishnan M.L., Mapelli M., Choi S., Tidor B., Cuny G.D., Musacchio A., Yeh L.-A., Kosik K.S. (2005). Defining Cdk5 Ligand Chemical Space with Small Molecule Inhibitors of Tau Phosphorylation. Chem. Biol..

[27] Fedorov O., Huber K., Eisenreich A., Filippakopoulos P., King O., Bullock A.N., Szklarczyk D., Jensen L.J., Fabbro D., Trappe J. (2011). Specific CLK inhibitors from a novel chemotype for regulation of alternative splicing. Chem. Biol..

[28] Soundararajan M., Roos A.K., Savitsky P., Filippakopoulos P., Kettenbach A.N., Olsen J.V., Gerber S.A., Eswaran J., Knapp S., Elkins J.M. (2013). Structures of Down syndrome kinases, DYRKs, reveal mechanisms of kinase activation and substrate recognition. Structure.

[29] Bosc N., Meyer C., Bonnet P. (2017). The use of novel selectivity metrics in kinase research. BMC Bioinform..

[30] Malumbres M. (2014). Cyclin-dependent kinases. Genome Biol..

[31] Juvale K., Stefan K., Wiese M. (2013). Synthesis and biological evaluation of flavones and benzoflavones as inhibitors of BCRP/ABCG2. Eur. J. Med. Chem..

[32] Klatt F., Leitner A., Kim I.V., Ho-Xuan H., Schneider E.V., Langhammer F., Weinmann R., Müller M.R., Huber R., Meister G. (2020). A precisely positioned MED12 activation helix stimulates CDK8 kinase activity. Proc. Natl. Acad. Sci. USA.

[33] Ashburner M., Ball C.A., Blake J.A., Botstein D., Butler H., Cherry J.M., Davis A.P., Dolinski K., Dwight S.S., Eppig J.T. (2000). Gene Ontology: Tool for the unification of biology. Nat. Genet..

[34] Consortium T.G.O. (2017). Expansion of the Gene Ontology knowledgebase and resources. Nucleic Acids Res..

[35] The UniProt Consortium (2025). UniProt: The Universal Protein Knowledgebase in 2025. Nucleic Acids Res..

[36] The UniProt Consortium (2019). UniProt: A worldwide hub of protein knowledge. Nucleic Acids Res..

[37] Berthold M.R., Cebron N., Preisach C., Burkhardt H., Schmidt-Thieme L., Decker R. (2008). KNIME: The Konstanz Information Miner. Data Analysis, Machine Learning and Applications. Studies in Classification, Data Analysis, and Knowledge Organization.

[38] Srinivasan B., Lloyd M.D. (2025). Quantitation and Error Measurements in Dose–Response Curves. J. Med. Chem..

[39] Lipinski C.A., Lombardo F., Dominy B.W., Feeney P.J. (1997). Experimental and computational approaches to estimate solubility and permeability in drug discovery and development settings. Adv. Drug Deliv. Rev..

[40] Veber D.F., Johnson S.R., Cheng H.-Y., Smith B.R., Ward K.W., Kopple K.D. (2002). Molecular Properties That Influence the Oral Bioavailability of Drug Candidates. J. Med. Chem..

[41] Chemical Computing Group Inc. (2026). Molecular Operating Environment (MOE).

[42] Saubern S., Guha R., Baell J.B. (2011). KNIME Workflow to Assess PAINS Filters in SMARTS Format. Comparison of RDKit and Indigo Cheminformatics Libraries. Mol. Inform..

[43] Pedregosa F., Varoquaux G., Gramfort A., Michel V., Thirion B., Grisel O., Blondel M., Prettenhofer P., Weiss R., Dubourg V. (2011). Scikit-learn: Machine learning in Python. J. Mach. Learn. Res..

[44] Roskoski R. (2026). Properties of FDA-approved small molecule protein kinase inhibitors: A 2026 update. Pharmacol. Res..

[45] RDKit: Open-Source Cheminformatics. http://www.rdkit.org.

[46] (2013). Glide.

[47] Berman H.M., Westbrook J., Feng Z., Gilliland G., Bhat T.N., Weissig H., Shindyalov I.N., Bourne P.E. (2000). The Protein Data Bank. Nucleic Acids Res..

[48] Madhavi Sastry G., Adzhigirey M., Day T., Annabhimoju R., Sherman W. (2013). Protein and ligand preparation: Parameters, protocols, and influence on virtual screening enrichments. J. Comput.-Aided Mol. Des..

[49] Friesner R.A., Banks J.L., Murphy R.B., Halgren T.A., Klicic J.J., Mainz D.T., Repasky M.P., Knoll E.H., Shelley M., Perry J.K. (2004). Glide: A New Approach for Rapid, Accurate Docking and Scoring. 1. Method and Assessment of Docking Accuracy. J. Med. Chem..

[50] Friesner R.A., Murphy R.B., Repasky M.P., Frye L.L., Greenwood J.R., Halgren T.A., Sanschagrin P.C., Mainz D.T. (2006). Extra Precision Glide: Docking and Scoring Incorporating a Model of Hydrophobic Enclosure for Protein–Ligand Complexes. J. Med. Chem..

[51] Baumli S., Lolli G., Lowe E.D., Troiani S., Rusconi L., Bullock A.N., Debreczeni J.É., Knapp S., Johnson L.N. (2008). The structure of P-TEFb (CDK9/cyclin T1), its complex with flavopiridol and regulation by phosphorylation. EMBO J..

[52] Petzold G., Fischer E.S., Thomä N.H. (2016). Structural basis of lenalidomide-induced CK1αdegradation by the CRL4CRBN ubiquitin ligase. Nature.

[53] Maiolica A., De Medina Redondo M., Schoof E.M., Chaikuad A., Villa F., Gatti M., Jeganatgan S., Lou H.J., Novy K., Hauri S. (2014). Modulation of the chromatin phosphoproteome by the Haspin protein kinase. Mol. Cell. Proteom..

[54] Bach S., Knockaert M., Reinhardt J., Lozach O., Schmitt S., Baratte B., Koken M., Coburn S.P., Tang L., Jiang T. (2005). Roscovitine targets, protein kinases and pyridoxal kinase. J. Biol. Chem..

