# Supplementary material for: An Experimentally Validated Structure-Based Virtual Screening Approach to Identify Nucleotide-Binding Protein Inhibitors as a New Source of Kinase Inhibitors
Source: Pharmaceuticals (Basel). 2026 Jul 23;19(8):1138. doi: 10.3390/ph19081138 (PMC13516363; doi:10.3390/ph19081138)
Supplement: Supplementary file 1 [file pharmaceuticals-19-01138-s001.zip › pharmaceuticals-4382650-supplementary.pdf]

Supplementary Information

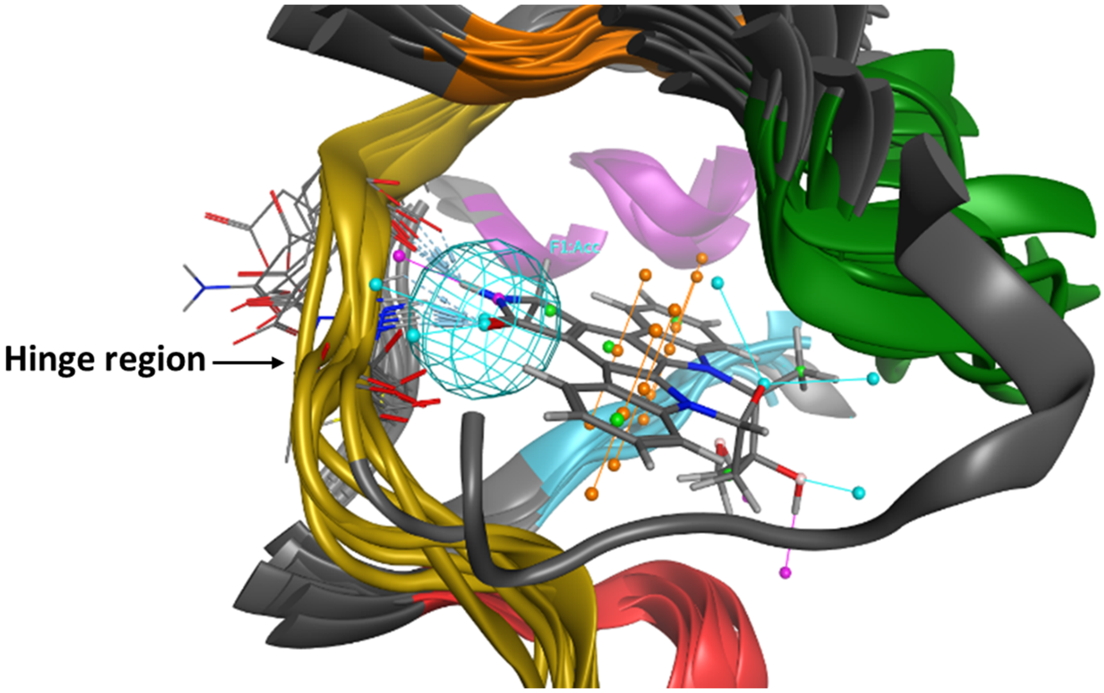

Figure S1. Pharmacophore designed for virtual screening. The pharmacophore contains a hydrogen bond acceptor with a radius of 2 Å (teal sphere). It was built using staurosporine ligand anchored to the aligned active sites of the different kinases AURKB (PDB entry 4AF3) [1], CDK2 (PDB entry 4EON) [2], CDK5 (PDB entry 3O0G) [3], CLK1 (PDB entry 2VAG) [4] and DYRK1A (PDB entry 2VX3) [5]. Hydrogen bond donors and their projected vectors are represented in pink dots, hydrogen bond acceptors and their projected vectors are in teal, aromatic rings and their projected vectors are in orange and hydrophobic features are in green.

Table S1. Percentage of remaining activity of 25 compounds obtained from the virtual screening for 10 protein kinases. Compounds that display a remaining activity less than 50 percent are highlighted in green.

ID	AURKB	CDK5	CDK9	CLK1	CSNK1A1	DYRK1A	HASPIN	PIM1	CDK2	RIPK3	AMBINTER ID	CHEMBL ID
1	94	86	86	87	112	52	100	95	115	106	Amb19772541	CHEMBL1299762
2	82	97	88	104	94	83	92	87	108	102	Amb2677038	CHEMBL1336054
3	96	97	109	77	64	98	80	102	105	92	Amb679230	CHEMBL1343463
4	63	96	87	76	82	100	79	94	100	97	Amb43693	CHEMBL104028
5	85	92	44	65	73	43	111	41	114	103	Amb17699749	CHEMBL1415852
6	94	91	103	84	95	81	93	92	67	95	Amb698858	CHEMBL1460302

7	133	87	85	56	99	74	66	110	111	102	Amb8285358	CHEMBL105101
8	79	86	38	31	89	23	41	76	83	107	Amb20651361	CHEMBL2420087
9	102	90	98	84	106	49	97	93	119	120	Amb2715018	CHEMBL1294
10	76	79	43	9	96	10	56	42	65	112	Amb619925	CHEMBL1193417
11	86	85	3	85	96	85	106	103	89	96	Amb22331350	CHEMBL595
12	29	84	50	28	97	45	98	93	76	107	Amb21928455	CHEMBL2420094
13	98	53	32	62	86	74	70	81	40	93	Amb2715209	CHEMBL31634
14	84	103	71	91	99	89	101	108	114	117	Amb21993684	CHEMBL1581396
15	110	92	86	53	75	73	98	131	111	89	Amb1199764	CHEMBL425560
16	85	91	97	90	289	53	93	94	104	104	Amb2406500	CHEMBL1894308
17	135	107	40	103	180	48	90	106	92	117	Amb616352	CHEMBL1982433
18	101	119	77	77	147	88	122	96	110	116	Amb20242383	CHEMBL52609
19	79	98	89	81	98	89	137	95	115	95	Amb8571860	CHEMBL2180118
20	72	130	113	90	116	108	107	162	122	94	Amb19913797	CHEMBL2180095
21	73	84	98	101	117	108	130	101	107	100	Amb353243	CHEMBL1977983
22	69	83	82	88	101	106	118	74	94	108	Amb4003200	CHEMBL276076
23	72	98	84	80	94	89	103	102	109	89	Amb535482	CHEMBL121
24	71	107	95	81	100	104	100	94	129	101	Amb663973	CHEMBL2180113
25	84	121	111	81	120	99	106	207	130	107	Amb738752	CHEMBL1257422

Table S2. Biological activity data of the nearest PKI neighbors attached to the different hits, compounds 8, 9, 10, 11, 12 and 13. Data were extracted from ChEMBL database.

Hit compound		Nearest PKI neighbor	Activity value (nM)	Activity type	Kinases target
	ChEMBL ID	ChEMBL ID			
8	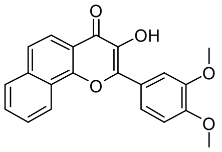 CHEMBL2420087	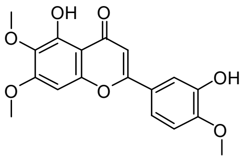 CHEMBL487402	794,33	Ki	Vascular endothelial growth factor receptor 2
			1000	Ki	Serine/threonine-protein kinase Aurora-A
			39,81	Ki	Serine/threonine-protein kinase Aurora-B

9	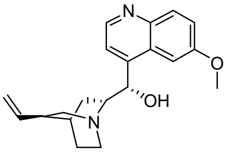 CHEMBL21578	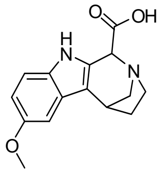 CHEMBL392876	90000	IC_{50}	MAP kinase-activated protein kinase 3
			200000	IC_{50}	c-Jun N-terminal kinase 2
			100000	IC_{50}	MAP kinase p38 alpha
			24000	IC_{50}	MAP kinase-activated protein kinase 5
			290	IC_{50}	MAP kinase-activated protein kinase 2
			200000	IC_{50}	MAP kinase-interacting serine/threonine-protein kinase MNK1
			200000	IC_{50}	Inhibitor of nuclear factor kappa B kinase beta subunit
			200000	IC_{50}	Ribosomal protein S6 kinase alpha 5
			200000	IC_{50}	Ribosomal protein S6 kinase alpha 5
10	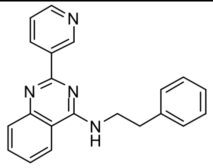 CHEMBL544818	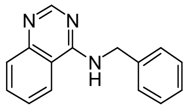 CHEMBL102726	320	IC_{50}	Epidermal growth factor receptor erbB1
11	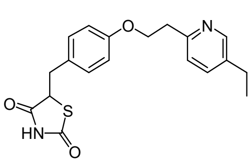 CHEMBL595	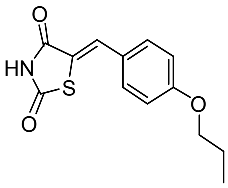 CHEMBL84558	150	IC_{50}	Serine/threonine-protein kinase PIM1
			20	IC_{50}	Serine/threonine-protein kinase PIM2
12	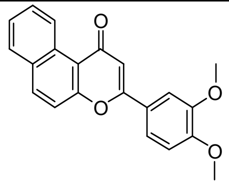 CHEMBL2420094	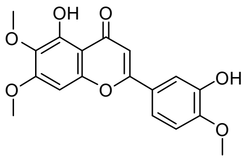 CHEMBL487402	794,33	Ki	Vascular endothelial growth factor receptor 2
			1000	Ki	Serine/threonine-protein kinase Aurora-A
			39,81	Ki	Serine/threonine-protein kinase Aurora-B
13	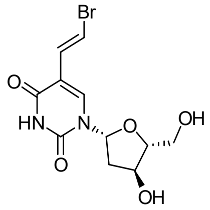 CHEMBL31634	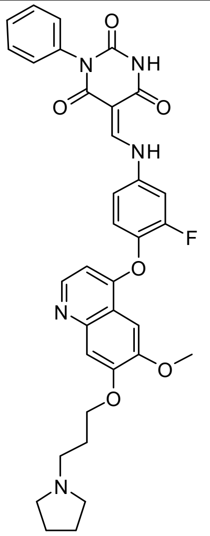 CHEMBL3116439	3,36	IC_{50}	Hepatocyte growth factor receptor

References

1. Elkins, J. M.; Santaguida, S.; Musacchio, A.; Knapp, S., Crystal Structure of Human Aurora B in Complex with INCENP and VX-680. *Journal of Medicinal Chemistry* **2012,** *55* (17), 7841—7848. doi:10.1021/jm3008954.
2. Echalier, A.; Cot, E.; Camasses, A.; Hodimont, E.; Hoh, F.; Jay, P.; Sheinerman, F.; Krasinska, L.; Fisher, D., An Integrated Chemical Biology Approach Provides Insight into Cdk2 Functional Redundancy and Inhibitor Sensitivity. *Chemistry & Biology* **2012,** *19* (8), 1028—1040. doi:10.1016/J.CHEMBIOL.2012.06.015.
3. Ahn, J. S.; Radhakrishnan, M. L.; Mapelli, M.; Choi, S.; Tidor, B.; Cuny, G. D.; Musacchio, A.; Yeh, L.-A.; Kosik, K. S., Defining Cdk5 Ligand Chemical Space with Small Molecule Inhibitors of Tau Phosphorylation. *Chemistry & Biology* **2005,** *12* (7), 811—823. doi:10.1016/j.chembiol.2005.05.011.
4. Fedorov, O.; Huber, K.; Eisenreich, A.; Filippakopoulos, P.; King, O.; Bullock, A. N.; Szklarczyk, D.; Jensen, L. J.; Fabbro, D.; Trappe, J.; Rauch, U.; Bracher, F.; Knapp, S., Specific CLK inhibitors from a novel chemotype for regulation of alternative splicing. *Chemistry & biology* **2011,** *18* (1), 67—76. doi:10.1016/j.chembiol.2010.11.009.
5. Soundararajan, M.; Roos, A. K.; Savitsky, P.; Filippakopoulos, P.; Kettenbach, A. N.; Olsen, J. V.; Gerber, S. A.; Eswaran, J.; Knapp, S.; Elkins, J. M., Structures of Down syndrome kinases, DYRKs, reveal mechanisms of kinase activation and substrate recognition. *Structure* **2013,** *21* (6), 986—996. doi:10.1016/j.str.2013.03.012.
